# Molecular Modeling Study of the Genotoxicity of the Sudan I and Sudan II Azo Dyes and Their Metabolites

**DOI:** 10.3389/fchem.2022.880782

**Published:** 2022-06-23

**Authors:** Rachelle J. Bienstock, Lalith Perera, Melissa A. Pasquinelli

**Affiliations:** ^1^ Fiber and Polymer Science Program, Wilson College of Textiles, North Carolina State University, Raleigh, NC, United States; ^2^ National Institute of Environmental Health Sciences, Research Triangle Park, Durham, NC, United States; ^3^ Forest Biomaterials, College of Natural Resources, North Carolina State University, Raleigh, NC, United States

**Keywords:** azo dyes, Sudan I, Sudan II, molecular dynamics simulations, DNA adducts, DNA damage, genotoxicity

## Abstract

Azo dyes are defined by the presence of a characteristic N=N group. Sudan I and Sudan II are synthetic azo dyes that have been used as coloring agents. Although animal toxicity studies suggest that Sudan dyes are mutagenic, their molecular mechanism of action is unknown, thus making it challenging to establish thresholds for tolerable daily intake or to understand how these molecules could be modified to ameliorate toxicity. In addition, dye metabolites, such as azobiphenyl and 4-aminobiphenyl, have been correlated with epigenetic alterations. We shed some light on the mechanisms of Sudan dye genotoxicity through a molecular modeling study of Sudan I and Sudan II dyes and two common metabolites interacting with DNA as adducts. The results suggest that all four adducts cause significant perturbations to the DNA helical conformation and structure; thus, it can be inferred that DNA repair and replication processes would be significantly impacted.

## Introduction

Azo dyes are a group of dyes that contain substituent groups connected with an N=N bond, and are commonly used as colorants in a variety of commercial and manufacturing industries, including for textiles and food. Many azo dyes are known to generate one or more aromatic amine metabolites, which can be found in commercial hair dyes, color additives, paints, and food colors. Many of these dyes are regulated compounds through a variety of environmental laws such as REACH. Animal toxicity studies indicate that some azo dyes and their metabolites possess genotoxic and carcinogenic properties (Tsuda et al., 2001; An et al., 2007; Al Reza et al., 2019; Jiang et al., 2020; Xing et al., 2021). However, the precise molecular mode of action of these compounds is unknown, although adduct formation with DNA is considered to be a factor. Adduct formation can cause DNA damage and can also affect DNA replication and/or transcription. While DNA adducts have been correlated with carcinogenicity in animal studies, many site-specific mutations arise due to the presence of such DNA adducts (Guengerich, 2006). The identification and characterization of a DNA adduct can provide evidence of the mechanism of genotoxicity and lead to legislative limits on exposure. Thus, the primary goal of this study is to obtain structural information to understand the mechanism of azo dye genotoxicity through their interactions and adduct formation with DNA and to look into the potential for DNA damage and thus identify potential repair mechanisms.

Sudan dyes are industrial synthetic azo dyes that are used in great quantities as colorants in plastics, printing inks, textiles, and consumer products, especially those that need a red or orange hue. In anaerobic conditions, azobenzene reductase can reduce azo dyes to amines. The use of Sudan dyes in food products is not permitted since they have been classified as mutagens (IARC, International Agency for Research on Cancer, European Parliament and Council Directive 94/36/EC). However, Sudan I [1-(phenylazo)-2-naphthol, C.I. Solvent Yellow 14] has frequently been identified as a contaminant and adulterant in chili and curry powders, turmeric, paprika, satay sauce, Italian sauces, saffron, palm oil, FD&C Yellow no. 6, D&C orange no. 4, and animal feed (Coulet et al., 2010; Fonovich, 2013), despite its classification by the IARC as a category 3 carcinogen.

Sudan dyes are also of significant interest to government environmental agencies since they can become significant contaminants not only in food but also in aquatic environment, and many are considered priority pollutants for investigate since little is known about their mode of action (Zanoni et al., 2013). In mammalian cell studies, Comet assays indicated that Sudan I exhibits mutagenic activity, and specifically that it can cause DNA strand breaking (An et al., 2007). In mouse models, Sudan I was reported to not only disrupt spindle organization and chromosomal alignment but also the destruction of mitochondrial functions, accumulating reactive oxygen species (Xing et al., 2021). Sudan I was also implicated in potentiating the genotoxicity of human carcinogen benzo [a]pyrene in mouse models (Dracinska et al., 2021). Between 2003 and 2005, over 600 food products containing Sudan dyes were recalled in the United Kingdom. Sudan I, II, and III, Sudan Brown RR, Red 7B, and Black B are classified as group 4 carcinogens, which means that there is inadequate or limited experimental evidence regarding their carcinogenicity. Sudan II is still permitted for use in cosmetics, although since 2003, it has not been permitted in food products. Sudan II, III, and IV were identified in Chinese lipsticks despite studies (Coulet et al., 2010) that have indicated that they can be absorbed through skin exposure. Although Sudan dyes are classified as mutagens based on animal toxicity studies, their molecular mode of action is unknown; thus, it is challenging to establish thresholds for tolerable daily intake or to understand how these molecules could be modified to ameliorate toxicity.

In addition, some metabolites of Sudan dyes, typically aromatic amines, have been correlated with epigenetic alterations (Chappell et al., 2016), and *in vivo* studies in rats and rabbits have indicated mutagenicity (Chappell et al., 2016). Sudan I can be enzymatically oxidized and/or reduced in metabolic pathways within the cell, namely hepatic and bladder enzymes yielding mutagenic metabolites (Cox and White, 2019); reductive cleaving can produce aniline and 1-amino-2-naphthol. Sudan Black B and Sudan III, commonly used for cell and tissue staining, metabolize to colorless carcinogenic amines in the liver. Sudan II can metabolize p-phenylenediamine and aniline. There is some evidence that Sudan I may be oxidized and activated metabolically by CYP450 enzymes to produce mutagenic and genotoxic products. Cytochrome P450 1A1 (CY1A1) catalyzes the oxidation of Sudan I to form a reactive metabolite, the benzenediazonium cation, leading to its ability to form DNA adducts and increasing its carcinogenicity (Tumbi et al., 2014). In a study evaluating the impact of exposure to Sudan dyes and their metabolites on human intestinal bacteria, it was reported that the dyes and their metabolites could inhibit the growth of some intestinal bacteria (Pan et al., 2012).

Compounds such as azo dyes and their metabolites form adducts with DNA when reactive intermediates form that then attack the DNA nucleobases. For instance, the metabolic pathway for Sudan I is induced when peroxidases oxidize the dye to form a radical species, and the radical then attacks the exocyclic amino group of guanines (G) to form a DNA adduct (Dračínský et al., 2009). The general mechanism for azo dye-DNA interactions is understood to be that the azo dyes and their metabolites form nitrenium ions, which are electrophilic DNA binders, that preferentially bind to either the G C8 or N2 atoms (Manderville and Wetmore, 2017). These Sudan I metabolite-DNA adducts have been identified *in vivo* in the liver of rats exposed to these dyes. In the bladder, peroxidases were observed to catalyze Sudan I metabolism which reacts with deoxyguanosine (dG), leading to the formation of 4-[(deoxy)guanosin-N2-yl] (Dračínský et al., 2009); this structure was used for the basis of this modeling study.

In the majority of cases, dG is the DNA nucleoside base that is modified by the formation of DNA-Sudan family adducts. The low oxidation potential of the 2′-dG makes it particularly susceptible to reactions. Several dG sites can form reactive adducts (including N2, O6, N1, N3, and N7); however, C8 is the preferential site for most toxins. Deoxyguanosine is modified, to a lesser extent, in DNA interactions with Sudan I in which case an 8-(phenylazo) G DNA adduct forms (Yun et al., 2020). Many studies in the literature have reported on DNA-aromatic amine adducts that target C8-dG and N2-dG.

In addition to DNA mutations due to the formation of DNA adducts, epigenetic alterations can also arise due to chemical exposure (Chung, 2016), particularly with compounds that form G adducts. Epigenetic alterations cause changes in gene expression. Research into whether epigenetic alterations are major contributing factors in cancer development is an increasing area of interest**.** Epigenetic alterations have already been demonstrated to occur with the polycyclic aromatic hydrocarbons benzo(a)pyrene and *anti*-BPDE-DNA (where BPDE is 7,8-dihydro-7,8-dihydroxy benzo [a]pyrene 9,10-oxide) which preferentially form G adducts and in particular G nucleotide with a methylated cytosine (C) adjacent to the G nucleotide (5-MeCpG) (Kathryn and Bailey, 2012). Adducts formed on 5-MeCpGs have different conformations than those formed on unmethylated CpGs, and this may impact the efficiency of adduct removal, transcription, and DNA replication. Methylation of cytosine at C5 in a CpG sequence context causes a conformational transition from a minor grove binding alignment in the unmethylated system during the benzo [a]pyrene diol epoxide-N2-G adduct formation to an intercalative base pair alignment with a concomitant displacement into the minor groove in the methylated system (Zhang et al., 2005).

The focus of this work is on adducts formed by Sudan I and Sudan II and the metabolites azobiphenyl and aminobiphenyl, which preferentially form G adducts (Stiborova et al., 1995). Histone H3K4 mono-methylation was previously observed to decrease when cells were treated with 4- aminobiphenyl (Chappell et al., 2016). In that study exposing human HepG2 cells to 4- aminobiphenyl, the expression of 27 miRNAs was three times higher in cells treated with 4-aminobiphenyl and 16 DNA repair-related genes were downregulated (Chappell et al., 2016). In addition, double-strand break repair defective Rad54 and Ku70 minus cells were determined to be more sensitive to damage from Sudan I (Ooka et al., 2016). Adding benzo [a]pyrene (BP) derivatives to C-5 methylated CpG dinucleotide steps (5-MeCpGs) in DNA resulted in conformational differences while creating mutational hot spots (Zhang et al., 2005).

There are only a few published experimental studies *in vitro* that report the structure of specific covalent azo-DNA or azo-metabolite adducts; typically, the structures are isolated with 32P-postlabeling assays in high-performance liquid chromatography and then identified using ^14^C labeled NMR studies, mass spectrometry (MS), or X-ray crystallography. For instance, Dracinsky et al. sought to isolate DNA or RNA Sudan I adducts using these approaches (Dračínský et al., 2009). A metabolite of Sudan I, Solvent Yellow 14 (1-(phenylazo)-2-hydroxynapththalene (CAS 842-07-9) was revealed to form guanosine adducts with DNA. Due to challenges in isolating and identifying these DNA adducts formed by these dyes and their metabolite, there is a lack of information available from experiments on the DNA damage caused by these adducts, which illustrates the need for molecular modeling.

The goal of this work is to use molecular mechanics (MM) calculations and molecular dynamics (MD) simulations to understand mutagenesis and carcinogenicity on a molecular level of these azo dyes and their metabolites by examining site-specific chemical adduct formation in DNA and the damage it could induce to DNA. Specifically, we performed 1 μs MD simulations using explicit water and evaluated a variety of characteristics, such as variations in major and minor groove widths measured by the phosphorus to phosphorus distances in the helix backbone, changes in DNA base pair orientations, alterations in hydrogen bonding between base pairs and glycosidic bonds (base to sugar bonding), and Watson–Crick versus Hoogsteen base pairing. The structural insights reported here on the damage that could be induced in DNA from adduct formation could also lead to an understanding of how these DNA adducts interact with the proteins in the DNA repair enzyme system. Thus, these modeling studies of DNA with adducts and lesions can play a strategic role in predicting and understanding the genetic impact of these toxicants.

## Computational Details

### Creating Molecular Models

**FIGURE 1:**
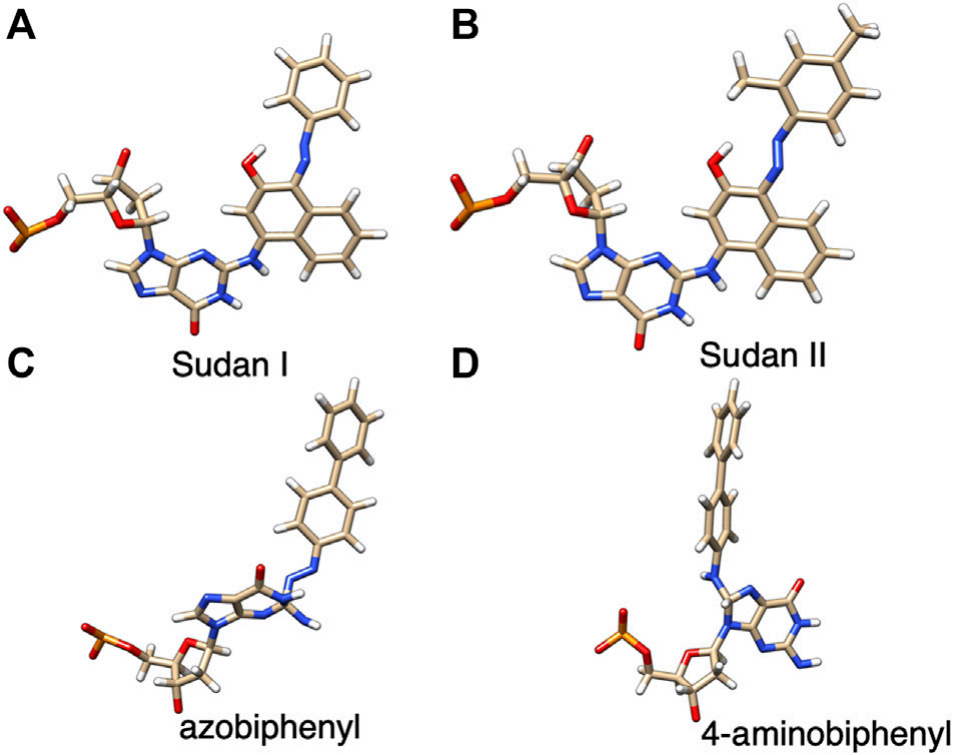
Structures of dG adducts with **(A)** Sudan I, **(B)** Sudan II, **(C)** azobiphenyl, and **(D)** 4-aminobiphenyl, where red, oxgyen; blue, nitrogen; brown, carbon; orange, phosphorus; and white, hydrogen.

The starting structures of the deoxyguanosine (dG) mononucleosides adducts with Sudan I, Sudan II, aminobiphenyl, and azobiphenyl are given in Figure 1. The connectivity of Sudan dye adducts to DNA for starting structures for the modeling studies was based on NMR structural studies {4-[(deoxy)guanosin-N2-yl] Sudan I adduct} (Dračínský et al., 2009). Zanonia et al. recently reported the formation of guanosine-Sudan stable adducts, which they identified using ESI/MS/MS techniques (Zanonia et al., 2013). They agreed with other findings that the C8 G position is the primary location of adducts due to its susceptibility to S_N_2 nucleophilic attack. The C8 G starting 4-aminobiphenyl DNA adduct structure was based on mass spectrometry (Means et al., 2003), although these were adenine rather than G adducts. The structure files in the *sdf* format for Sudan I (Solvent Yellow 14, C16H12N2O, CAS 842-07-9 1-Phenylazo-2-naphthalenol), Sudan II {Solvent Orange 7, C18H16N2O, CAS 3118-97-6, 1-[(2,4-Dimethyl Phenyl) azo]-2-naphthalenol}, azobiphenyl, and 4-aminobiphenyl were obtained and verified from both PubChem and ChemSpider. The Gaussview/Gaussian-16 (Frisch et al., 2016) software package was used to optimize structures and to derive ESP charges for the dye molecule alone and dye-DNA adduct molecules at the HF/6-31G (d, p) level.

**FIGURE 2:**
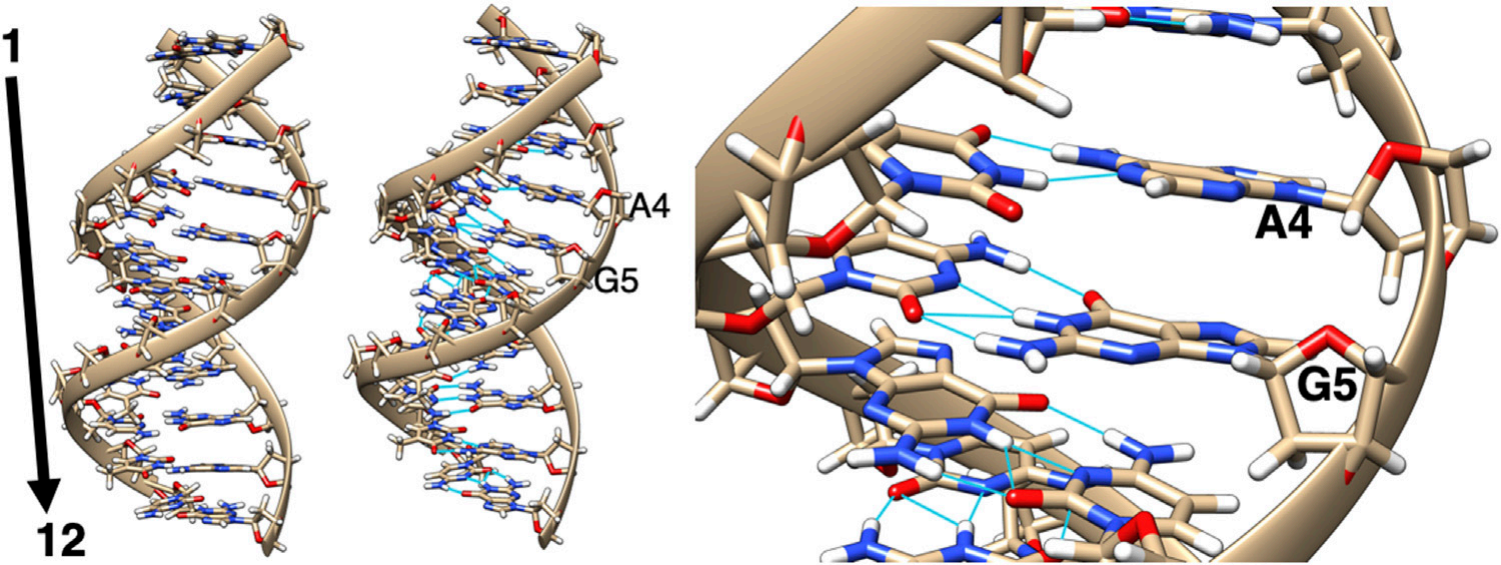
Two views of the minimized structure (left and middle) of the unaltered DNA dodecamer with a sequence of 5′-D (-GP-AP-GP-AP-GP-CP-GP-CP-TP-CP-TP-C)-3′, with the 5th G residue (zoomed in, right) being modified in the adduct formation.

The DNA sequence selected for these adduct modeling studies is the DNA dodecamer containing the 7-aminomethyl-7-deaza-2′-deoxyguanosine adduct. We used the solution NMR structure of this DNA dodecamer, which was obtained from the PDB file, 2LIA, and was further used as the template DNA structure (Szulik et al., 2013). As depicted in Figure 2, this DNA dodecamer duplex 5′-D (-GP-AP-GP-AP-GP-CP-GP-CP-TP-CP-TP-C)-3′), where A is adenine, G is guanine, C is cytosine, and T is thymine, was used as the base model DNA structure for adduct-modification, with the 5th G residue modified to obtain the adducts where the 7-aminomethyl-7-deaza-guanine group was replaced with Sudan I-, and Sudan II-, azobiphenyl-, and aminobiphenyl-guanine groups. A central residue was selected to avoid DNA end effects. All of these systems contained only one Sudan dye with azobiphenyl or aminobiphenyl residues bound to the 5th G nucleotide in the center of the dodecamer helix. After creating these dodecamer DNA adduct structures, they were first minimized using the Sander module of Amber.16 (Case et al., 2016) *in vacuo* with the AMBER force field, including the modified force field parameters to accommodate the ligand. The FF14SB and DNA.OL15 force fields were used for standard nucleotides with parm10.dat and gaff2 and the customized force field for lesions. Explicit water molecules were represented by the TIP3P model and Na+ counter ions by the standard Amber force field parameters listed for ions.

### MD Simulations

Various modules of Amber-16 and AmberTools software packages (Case et al., 2016) were used for setting up and running all DNA adduct simulations. A leap library file was created for each dye adduct molecule with a modified force file containing custom Amber parameters and the ESP charges calculated from Gaussian.16. The Amber antechamber program was used to ensure that there was correct parameterization within Amber for each G dye adduct.

Solvated MD simulations were performed on each system to determine conformational perturbations due to dye-adduct formation on the DNA dodecamer duplex. Each system was first solvated with a water box using TIP3P water molecules extending at least 15 Å from the closest DNA atom to the boundary of the box and charge-balanced with sodium counterions, minimized and subjected to first constrained dynamics, followed by unconstrained solvated MD for 1 μs for each system in the constant pressure-constant temperature (NPT) ensemble with T = 300 K and *p* = 1 atm. The solvated systems typically contained on the order of 32,000–35,000 atoms. Box dimensions were typically 70 × 82 × 83 Å^3^. Coordinates used for the analysis were extracted at every 1 ns from each MD trajectory. The same simulation protocol was followed with a control DNA dodecamer, without a dye or metabolite deoxyguanine adduct. Identical MD trajectories were calculated on this unmodified DNA duplex as a control to quantify conformational changes due to the coordination of the dye molecules in the DNA adduct simulations.

### Analysis of MD Simulation Trajectories

To perform conformational analysis, the resulting coordinates collected at every 10 ns from every MD trajectory were saved in PDB format to characterize the structural perturbations in the DNA helix due to the presence of adducts. DNA base interactions and hydrogen bonding patterns were analyzed using the program 3DNA (Lu and Olson, 2003). Changes were monitored in DNA conformations and structures due to the lesion present, including minor groove/major groove size distortions, B-DNA helix parameters, and Watson–Crick base pairing and H bonding disruptions. Local base-pair parameters (shear, stretch, stagger, buckle, propeller, and opening) and local base-pair step parameters (shift, slide, rise, tilt, roll, and twist) were evaluated and compared for all structures. Using 1,000 samples collected at each ns over the µs simulations, the interaction energies between the two DNA strands were calculated using the MM/GBSA (generalized Born surface area) protocol in Amber.16 with the default parameters and at a salt concentration of 100 mM.

## Results

For the purpose of this MD simulation study, Sudan I and II azo dyes and the azo dye metabolites aminobiphenyl and azobiphenyl were selected as DNA adducts. Azobiphenyl and aminobiphenyl are frequent metabolites of azo dyes and also are the base compounds for many fabric dyes; these aromatic amines have been observed to directly form DNA adducts [i.e., N-(deoxyguanosin-N2-yl) -4-azobiphenyl] (Hatcher and Swaminathan, 2002; Chappell et al., 2016). Metabolites of many arylamines have been demonstrated to react *in vitro* and *in vivo* with C-8 of G (Babu et al., 1992; Zenser et al., 1998). Zanonia and coauthors (Zanonia et al., 2013) recently identified the formation of guanosine-Sudan stable adducts using electrospray ionization with MS/MS techniques and confirmed other findings that the primary location of adducts formation is the G C8 position due to its susceptibility to S_N_2 nucleophilic attack.

The connectivity of Sudan dye adducts to DNA in the starting structures for the previously reported modeling studies was determined from NMR structural studies of 4-[(deoxy)guanosin-N2-yl] Sudan I adduct (Dračínský et al., 2009). We chose to set up and validate the initial molecular models using the structure that has been previously determined through mass spectrometry and NMR structural analysis because of the explicit chemistry of DNA base adduct formation in this model. Biphenyl-modified G have been previously modeled and reported in the literature (Shapiro et al., 1995), which indicated that the aminobiphenyl was bonded to the G C8 position in the DNA adduct; modeling included a benzidine adduct with a variation of all DNA trimers and then from those trimers, they modeled DNA nonamers. However, in our study, the dye molecules and derivatives are covalently bonded to the C2 or N2 position of G, and the initial DNA structure was based on the reported NMR work by Hatcher and Swaminathan (2002).

Biphenyl molecules in the minor groove caused greater DNA deformation, loss of base planarity and base-pair hydrogen bonding disruption (i.e., G did not hydrogen bond to opposite C), whereas phenyl bound to G in the major and minor groove in conformations that were competitive in stability. In the major groove, biphenyl molecules tend to not interfere with G hydrogen bonding. In the minor groove, Watson–Crick base pairing was destroyed, and the modified base was in the *syn* conformation. This base pairing would explain the frequent G to T and G to C transversions observed in the NMR studies of a 15mer DNA with aminobiphenyl adducts (Cho et al., 1992).

The selection of the DNA sequence is significant as the sequence context of the DNA oligomer can affect the conformation, insertion, and interactions of the adducts. We chose a DNA dodecamer containing the 7-aminomethyl-7-deaza-2′-deoxyguanosine adduct as it is a common basic DNA double-stranded helix used for modeling DNA conformations and adduct conformational perturbations. It is a standard B-DNA right-handed helix with Watson–Crick hydrogen-bonding interactions between the purines (A and G) and pyrimidines (T and C), which leads to A:T and G:C pairs. This pairing between complementary strands requires the nucleobases to adopt the *anti*-conformation of the glycosidic bond, which is defined by the dihedral angle c = 180 ± 90° (+[O4 ´C1 ´N9C4] for purines and + [O4 ´C1 ´N1C2] for pyrimidines) (Szulik et al., 2013). This dodecamer duplex, 5′-D (*GP*AP*GP*AP*GP*CP*GP*CP*TP*CP*TP*C)-3′), was used as a basic model DNA structure for adduct-modification at the 5th G residue.

One of the most significant analyses that aids in understanding the biological impact of the DNA adduct is the *anti*/*syn* conformational preferences of the DNA adduct base. Many damaged DNA bases, with adducts or lesions, form a *syn* glycosidic (base-sugar) bond and Hoogsteen hydrogen bonding base pairing, with a dihedral angle χ of 0° to ±90° degrees (from −90° to +90°) and an intra or extra helical DNA adduct base. Extrahelical adducts are in the major or minor grove, which contrasts with the standard B-DNA *anti* glycosidic (base-sugar) bond, and Watson–Crick base pairing with a dihedral angle χ of +90° to +180° or –90° to –180° (or 180°–270°) (Wilson et al., 2016). In addition, we calculated all other helix measurements and changes in base-pair parameters for all structures. Twist, tilt, roll, and slide measure the relative orientation between two successive base pair steps and thus give a representation of local overall DNA helical deformations. Twist represents the angle of the relative rotation between two successive base pairs around the helical axis. Roll and tilt define the angular deformations between two base pair planes along the long and short base pair axes, respectively. The roll angle opens up the minor groove side, and the tilt angle opens up the base pair steps in the direction of the phosphate backbone. Rise, slide, and shift define the translational displacement of neighboring base pairs. Shear, stretch and opening characterize hydrogen bonding features. Shear and stretch define the relative angular offset of the bases in the base plane. The opening is the angle between the 2 x-axes with respect to the normal of the base plane. Buckle, propeller, and stager describe the non–planarity of a given base. Based on the analysis by Lu and Olson (Lu and Olson, 2003) with 3DNA (Lu and Olson, 1999), the average values for B-DNA from high resolution crystal structures are reported as follows: buckle = 0.5 ± 6.7°, propeller = −11.4 ± 5.3°, opening = 0.6 ± 3.1°, shear = 0.00 ± 0.21 Å, stretch = −0.15 ± 0.12 Å, stagger = 0.09 ± 0.19 Å, tilt = -0.1 ± 2.5°, roll = 0.6 ± 5.2°, twist = 36.0 ± 6.8°, shift = 0.02 ± 0.45 Å, slide = 0.23 ± 0.81 Å, and rise = 3.32 ± 0.19 Å. Parameters obtained from the 3DNA analysis were calculated for both the unaltered DNA dodecamer and dye-bound DNA dodecamers as averages from various time points along the 1 µs MD trajectories.

**FIGURE 3:**
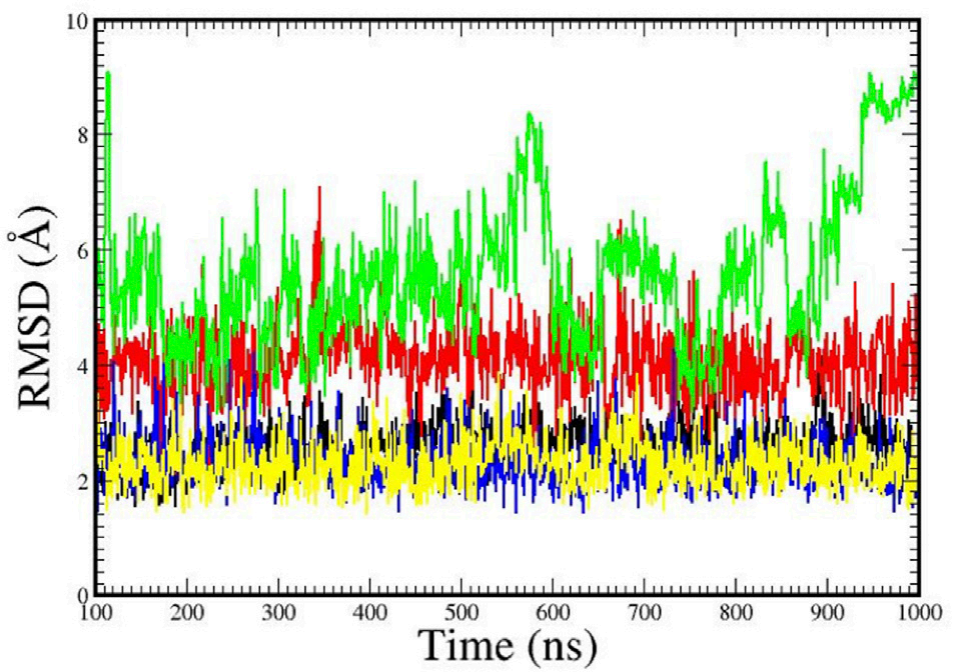
Root mean squared deviations (RMSDs) in the structures over the 1 µs time of the MD simulation trajectory. Legend: Adduct-free DNA (black); DNA with the aminobiphenyl adduct (green); DNA with the azobiphenyl adduct (red); DNA with the Sudan I dye adduct (yellow); DNA with the Sudan II dye adduct (blue).

Figure 3 illustrates the root-mean-square-deviations (RMSDs) for the structures over the 1 µs MD trajectories. The DNA-dodecamer structure with the aminobiphenyl adduct, shown in green, exhibited the greatest overall structural changes, with structures at the end of the MD trajectory displaying an RMSD in molecular coordinates greater than 8 Å. Examination of this structure illustrated a complete opening up of the double helix structure over the course of the trajectory. The structure with the azobiphenyl adduct, the RMSD shown in red, was approximately a consistent 4 Å over the course of its 1 µs MD trajectory. The Sudan dye adducts, which fit into the groove of the helix, had the smallest RMSDs, suggesting that the structure is comparable to that of the adduct-free DNA dodecamer.

### Confirmation of Unaltered DNA structure Model

**FIGURE 4:**
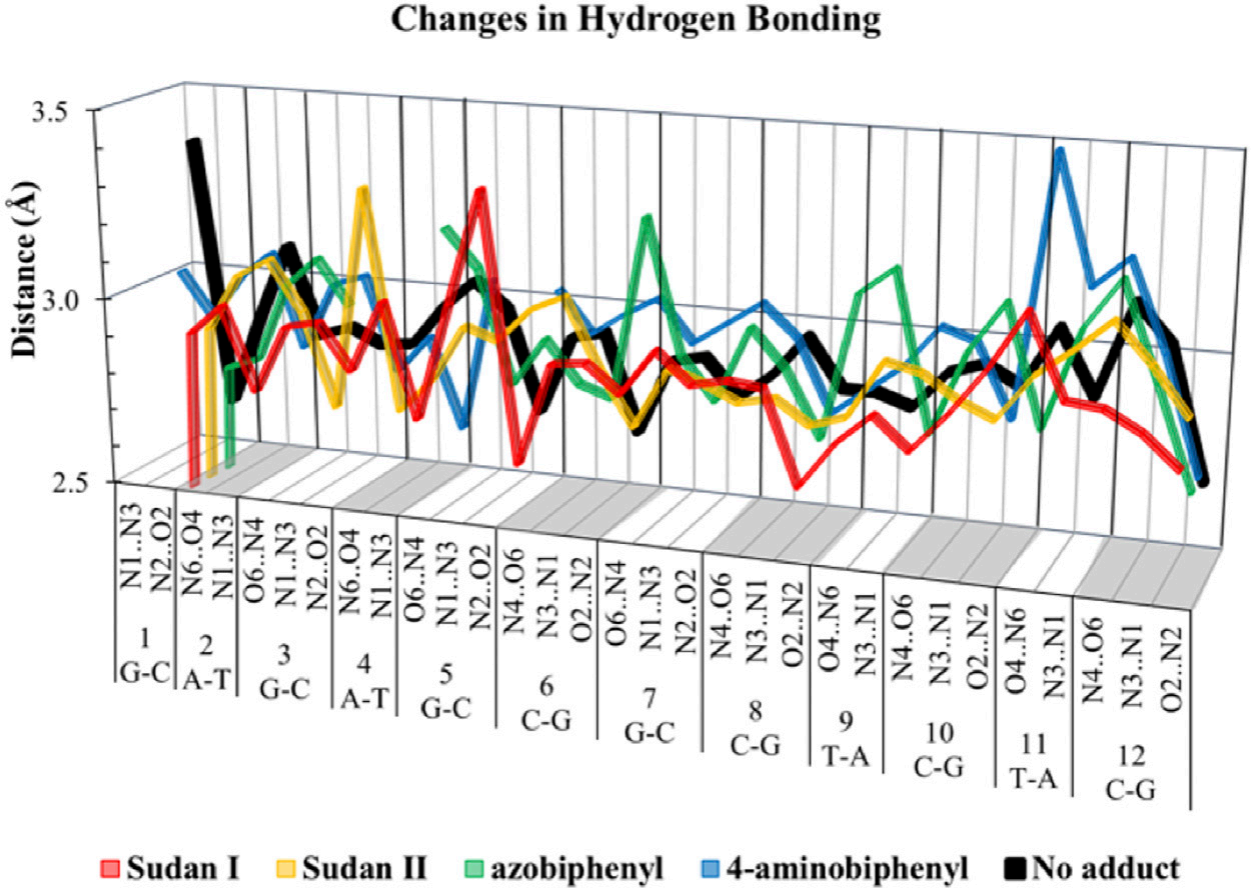
Length of various hydrogen bonds for unaltered DNA dodecamer (black) and adducts with Sudan I (red), Sudan II (orange), azobiphenyl (green), and 4-aminobiphenyl (blue).

**FIGURE 5:**
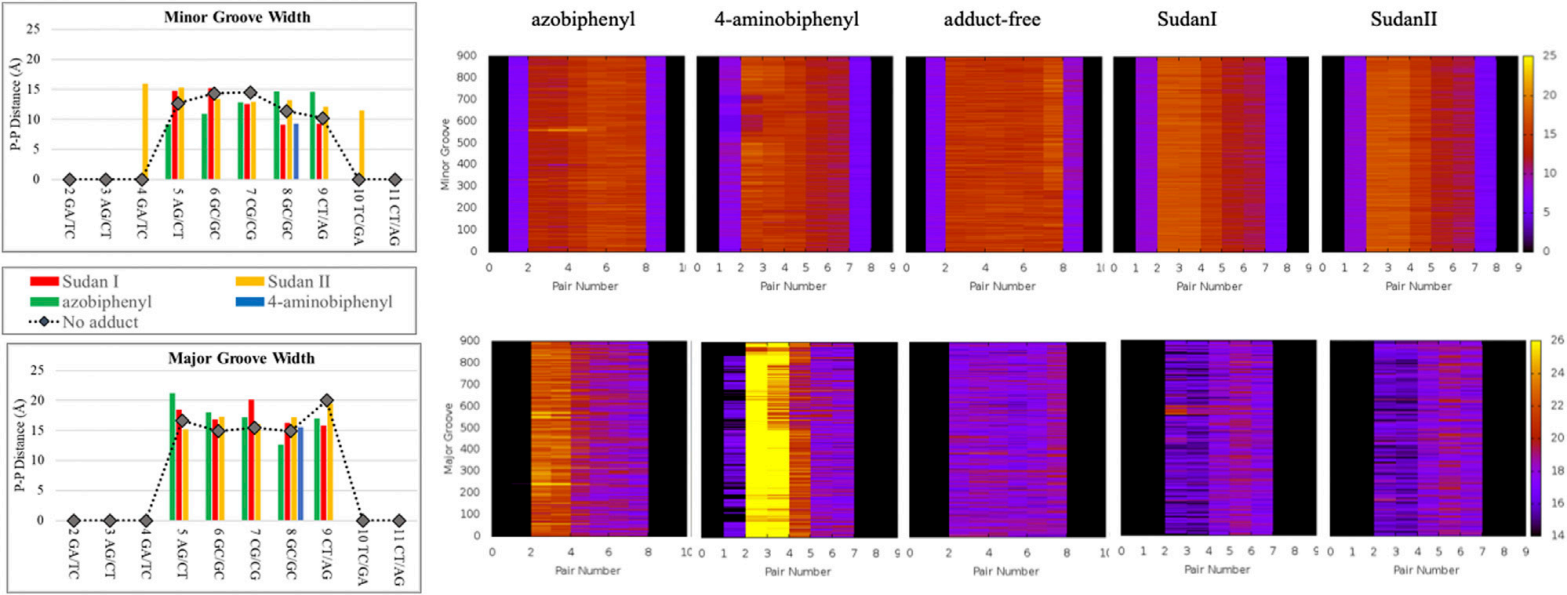
The P-P distances (in Å) of minor groove (top) and major groove (bottom) after 100 ns of MD simulations. The unaltered DNA dodecamer (black lines) and adducts with Sudan I (red), Sudan II (orange), azobiphenyl (green), and 4-aminobiphenyl (blue). Note that for the 4-aminobiphenyl adduct, only the 7GC/GC pair is quantified due to the disruption of the B-DNA helix between other base pairs. The heat maps illustrate the changes in minor and major grooves over the 1 µs trajectory. In the heat maps, the z-axis (or the scale on the right) represents the P-P distance in Å, and the y-axis (on the left) represents the time in ns (starting from 100 ns). The base pair numbers are across the x-axis.

**FIGURE 6:**
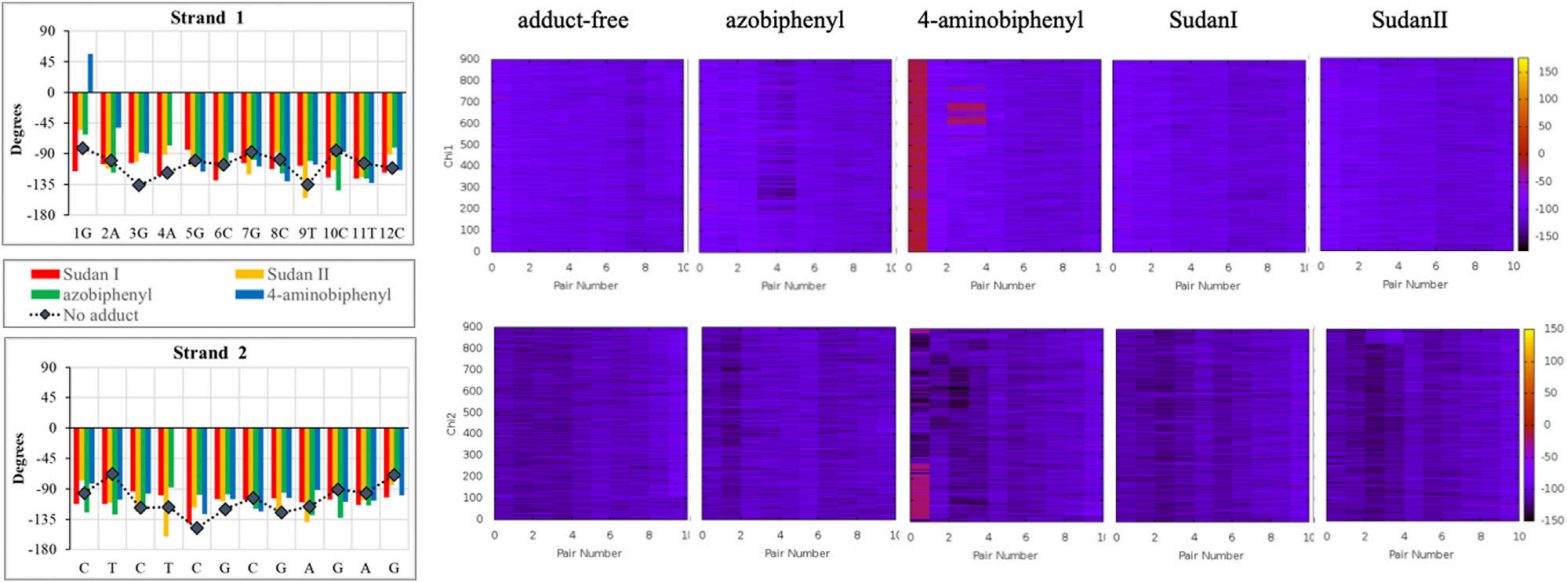
The χ angle variation in the unaltered DNA dodecamer (black lines) for each strand in the double helix after 100 ns of MD simulations and how those angles varied upon adduct formation with Sudan I (red), Sudan II (orange), azobiphenyl (green), and 4-aminobiphenyl (blue). The heat maps illustrate the changes in the χ angle on each of the DNA strands over the 1 µs trajectory. In the heat maps, the z-axis (on the scale on the right) represents the χ angle measurement in degrees, and the y-axis (on the left) represents the time in ns. The base pair number is across the x-axis.

**FIGURE 7:**
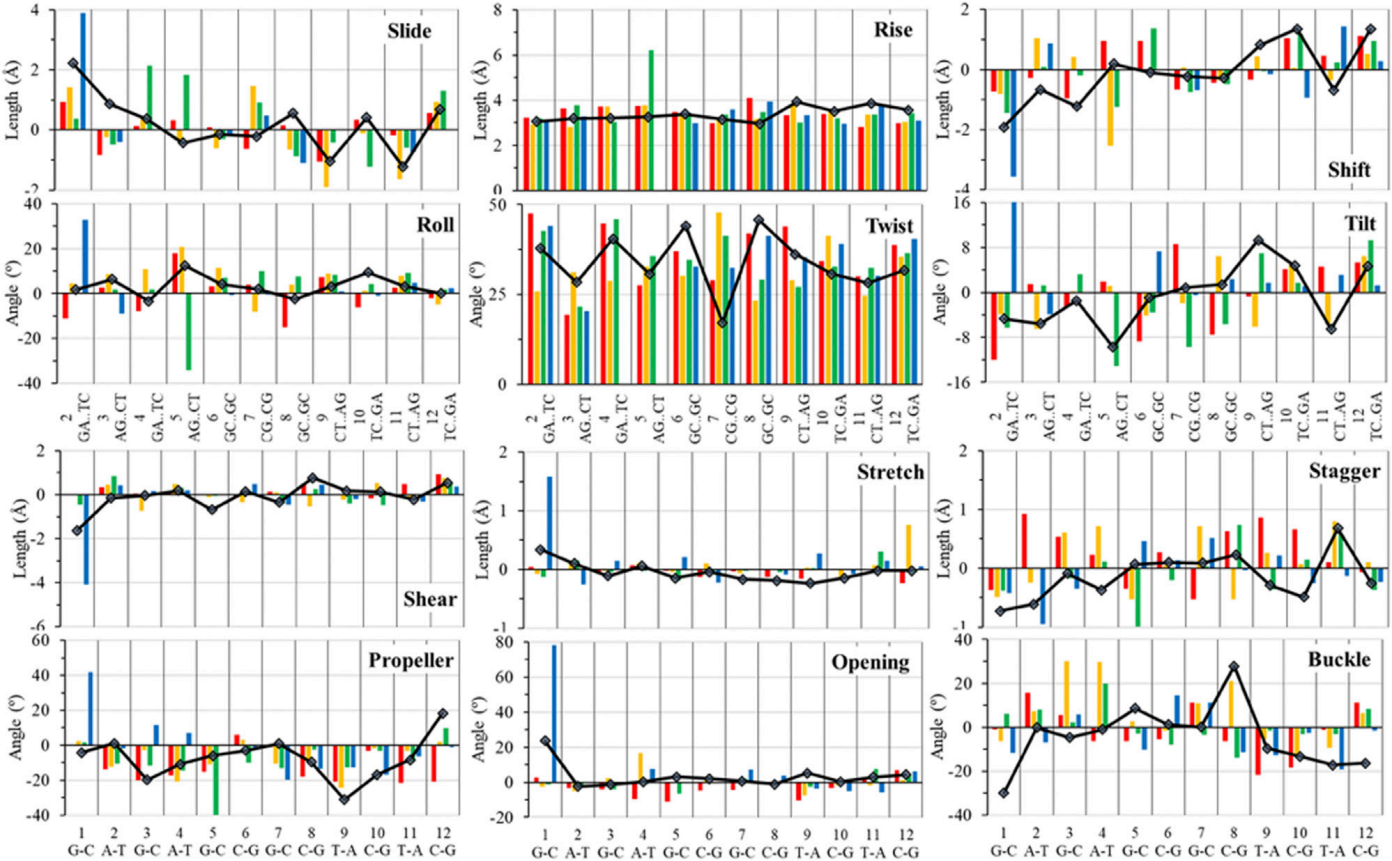
Top 6 panels represent the measure of local DNA helical deformation (calculated using the program 3DNA) with the following base pair parameters: slide, shift, tilt, roll, rise, and twist. The bottom six panels represent the measure of local DNA helical deformation with the following base pair parameters: shear, buckle, stretch, propeller, stagger, and opening. All structures were analyzed after 100 ns of MD simulations; the color scheme is the same as in left of Figure 6.

In over 1 µs of aqueous solvated MD simulations, the unaltered DNA dodecamer structure (without adduct, Figure 2), retains its B-DNA helix form and Watson–Crick H bonding (black line in Figure 4). 3DNA analysis indicates the initial minimized structure is B-DNA; however, the 5th, 6th, and 7th base pairs vary from B-DNA form after 50 ns but return to the B-DNA form after 70 and 80 ns. The changes in hydrogen bonding observed over the entire 1 µs MD simulation trajectory are summarized in Supplementary Figure S1. However, over the simulation time, the adduct-free DNA dodecamer structure is observed to exhibit minor variations in the major and minor groove width, which are measured as phosphorus to phosphorus distances and carbon to carbon distances in the helix backbone. The black line of Figure 5 (left) indicates that the minor grove is in the range of 11–14 Å and the major groove of 16–21 Å, consistent with B-DNA. Chi (χ) angles (black line in Figure 6) for bases vary, although largely fall within the −90 to −140 *anti* conformation. There are a few exceptions where there are nucleosides with χ angles in the *syn* range in the DNA dodecamer that occurred during the MD trajectory; however, *syn* conformations are not maintained. As observed in Figure 7, values for the helix parameters after the first 100 ns of the trajectory in the middle of the helix for the unaltered DNA dodecamer for shift, slide, rise, tilt, roll, shear, stretch, propeller opening, and stagger (bp5 GC/GC and bp6 CG/CG) agree with those anticipated for a B-DNA helix. Heat maps for the helical parameters for the entire 1 µs trajectory are given in Supplementary Figure S2. Thus, the dodecamer largely retains parameters for a B-DNA helix. The rest of the features in Figures 4–7 will be discussed below in detail.

### Changes to DNA Structure With the Sudan I Adduct

**FIGURE 8:**
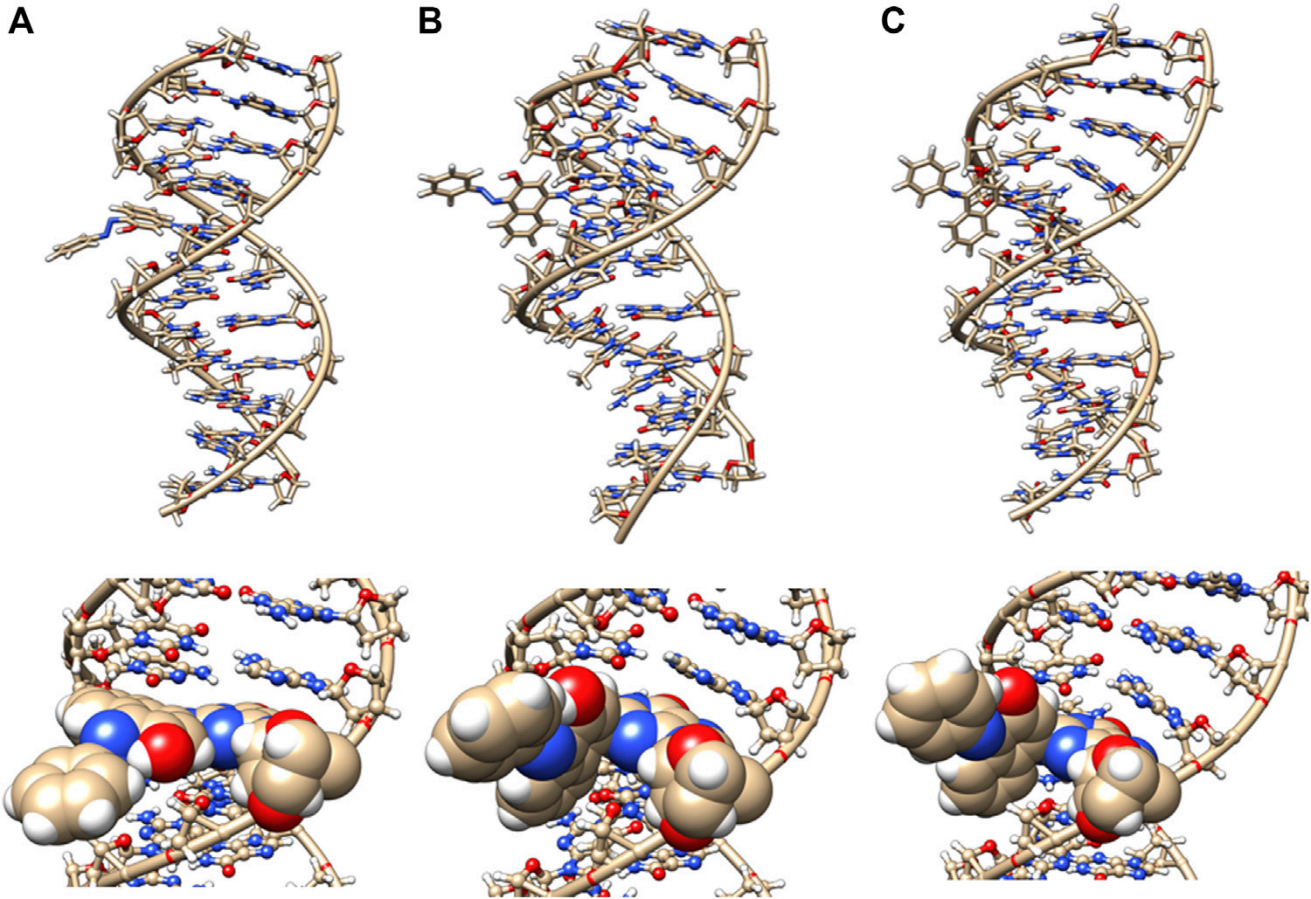
DNA dodecamer with Sudan I dye adduct. **(A)** Initial minimized structure, **(B)** at 100 ns of MD simulation, and **(C)** at 1 µs. The bottom row is a zoomed-in version of the adduct region.

In the initial minimized dodecamer DNA structure with a 5-dG- Sudan I adduct (Figure 8A), the adduct binds in the B-DNA minor groove and disturbs and distorts Watson–Crick base pairing and hydrogen bonding. The 4th base pair, prior to the Sudan adduct, exhibits significant helix distortion. The disruption in DNA structure appears to be annealed and resolved around 100 ns of MD simulations (Figure 8B), and hydrogen bonding and Watson–Crick base pairing are restored to the 5-dG Sudan I DNA dodecamer. The structure around 1 µs (Figure 8C) illustrates a further distance between base pairs.

In addition, around 50 ns of MD simulations, the DNA dodecamer with the Sudan I adduct begins to visit conformations with significant perturbations from the standard B-DNA helix. The structure at 70 ns has several bases (3G, 4A) with χ angles less than −90, indicating a *syn* glycoside bond; and at 80 ns the 4AG/CT and 5 GC/GC base pairs indicate a TA-like helix structural intermediate of A and B. After 100 ns, the center section of the adducted-dodecamer is no longer a B-DNA helix, and the 5G adducted base has a χ angle = −84° (red bars in Figure 6), characteristic of a *syn* glycosidic bond. As observed in Figure 4 (red line), the hydrogen bonding distances involving the adducted 5th GC base pair are greater than the unaltered DNA dodecamer. As indicated in Figure 5 (red bars), the phosphate to phosphate minor grove distances are larger for the Sudan I adduct than the unaltered DNA dodecamer in the 5th bp (AG/CT), near the lesion, 14.8 Å in the Sudan I adduct compared with 12.7 Å. In Figure 7 (red bars), the twist, roll and tilt measurements of the overall distortion in the DNA helix, due to adjacent base pair angle distortions, exhibit significant changes between unaltered DNA and the adduct (Sudan) damaged DNA. Values in the middle of the helix (bp4 AG/CT) and bp5 GC/GC and bp6 CG/CG for adducts differ significantly from idealized B-DNA crystal structure values and those for the unaltered DNA-dodecamer. Values around the adduct [the GC/CG (bp5) and GC/CG (bp6) values] for the lesions differ significantly from the helix without a lesion and from the average values reported in the literature for B-DNA. The twist value around the CG/CG lesion exhibits significant variation in the twist–relative orientation of the two stacked base pairs relative to the adduct-free DNA dodecamer. The tilt values for Sudan I adduct at the AG/CT base-pair near it are significantly different from others. Stagger for 3GC, 4AT, and 5GC near the lesion is significantly different for Sudan I. For propeller values, Sudan I differs at 7GC and 8GC.

### Changes to DNA Structure With the Sudan II Adduct

**FIGURE 9:**
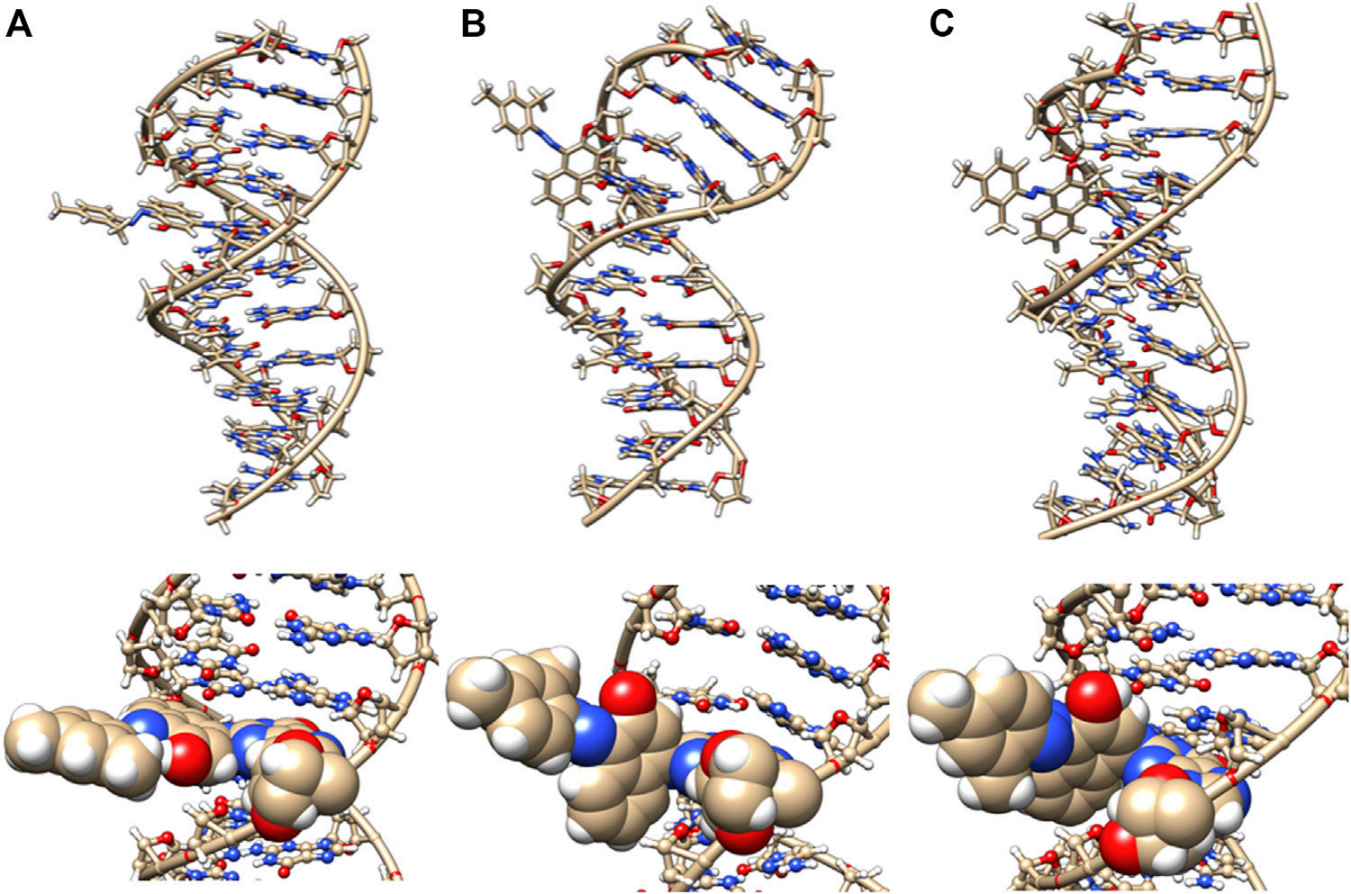
DNA dodecamer with Sudan II dye adduct. **(A)** Initial minimized structure, **(B)** at 100 ns of MD simulation, and **(C)** at 1 µs. The bottom row is a zoomed-in version of the adduct region.

The initial minimized dodecamer DNA structure with a 5-dG-Sudan II lesion (Figure 9A) exhibits disruption of the B-DNA helix and H bonding base-pair pattern. The B DNA helix is disrupted at the 4th dA-dT base pair, and the 4th base pair minor groove is 15.1 Å compared to 13.5 Å in the adduct-free DNA. All minor groove and major groove distances are larger than the adduct-free DNA dodecamer helix. The phosphate-phosphate groove measurements for the 4th and 5th base pairs, after 100 ns of dynamics (Figure 9B), are 7.1 Å and 7.2 Å, respectively, and only slightly larger than those for the free DNA dodecamer, which are 6.7 Å and 6.8 Å, respectively (Figure 5, orange bars). However, after 80 ns, the majority of the base pairs no longer fulfill the criteria for a B-DNA base pairing. After 100 ns, some of the damage due to the adduct is annealed, and only the 4AG/CT and 9TC/GA no longer fulfill the B-DNA helix criteria for base pairing. All χ values in Figure 6 (orange bars) are in the *anti* glycosidic bond range, with the exception of 8C, which is −88, and the end first residue 1G (−55), indicating that the end of the helix may be fraying or degenerating; note that there are often end effects and distortion in the end base pairs during the MD simulations of DNA. The χ angles were otherwise well maintained during the dynamics. In Figure 7 (orange bars), the tilt values for Sudan II at the AG/CT base pairs near it are significantly different from the rest. The shear differs significantly near the lesion base pairs, 3GC and 5GC. Stagger for 3GC, 4AT, and 5GC near the lesion are also significantly different for Sudan II. Buckle differs significantly near the adduct at 3GC and 4AT. For propeller, Sudan II differs at 7GC and 8GC.

Figure 7 provides the local helical parameters for DNA both with and without the Sudan adducts. Rise is observed to be much smaller in DNA in the presence of Sudan adducts and has some variance, as opposed to the adduct-free DNA. Roll is similarly variable in the base pairs just around the lesion, and the angle is much larger than in the unperturbed DNA. The twist parameter is much larger in modified DNA, particularly in the base pair directly above the lesions. The tilt angle is much larger and positive in modified DNA as opposed to the unperturbed DNA dodecamer, and again more variable in base pairs above the adduct. Slide is smaller and slightly negative in modified DNA compared to the unperturbed one. Overall, the minor groove distance is smaller in modified DNA, particularly around the lesion, and the major groove distance is smaller in modified DNA, particularly in base pairs above the lesions.

### Changes to DNA Structure With the Azobiphenyl Adduct

**FIGURE 10:**
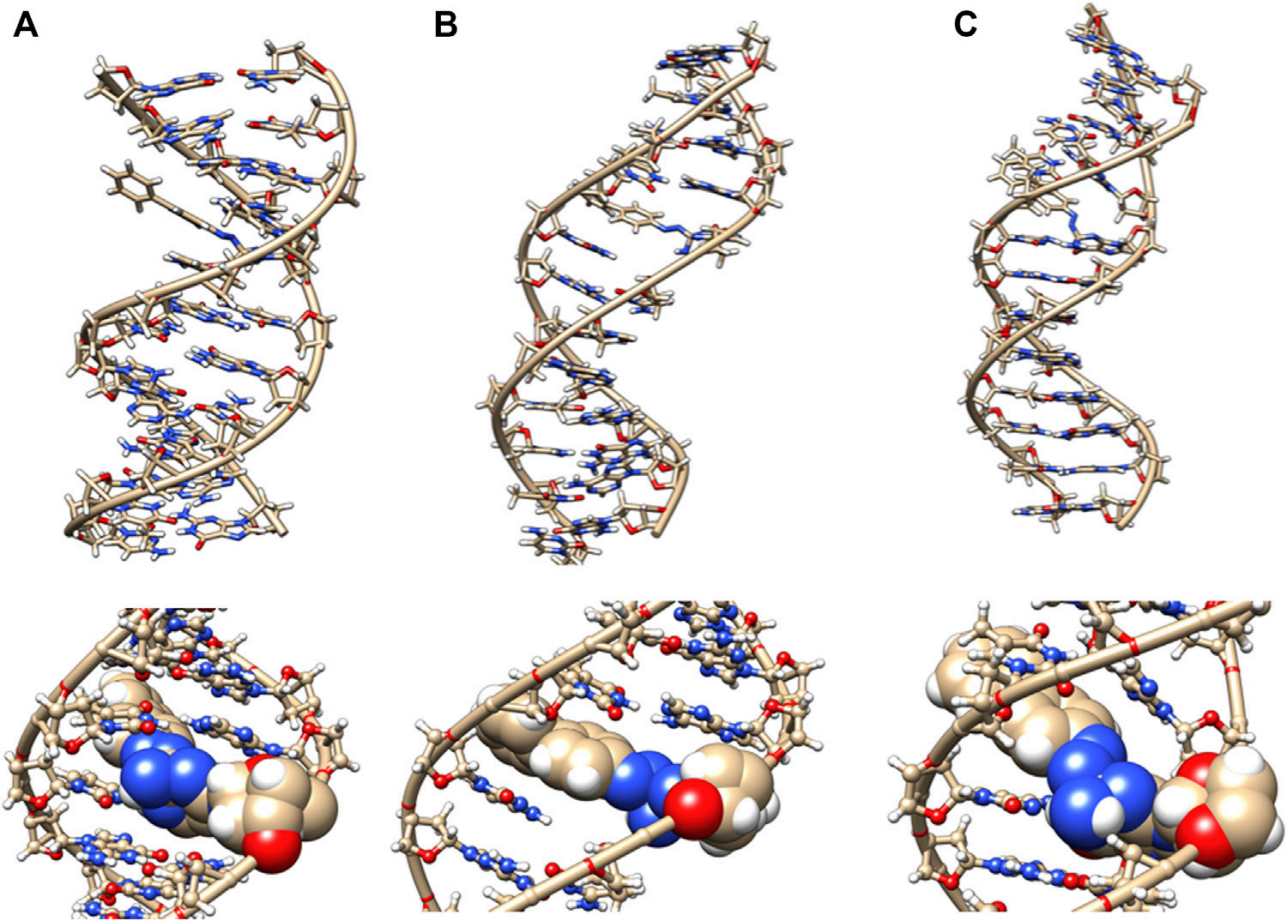
DNA dodecamer with azobiphenyl dye adduct. **(A)** Initial minimized structure, **(B)** at 100 ns of MD simulation, and **(C)** at 1 µs. The bottom row is a zoomed-in version of the adduct region.

The azobiphenyl DNA-adduct causes a small distortion to the diameter of the helix phosphate to phosphate minor groove diameter (Figure 5, green bars). The major groove diameter is distorted with the original adduct insertion but is annealed to a small diameter distortion after 100 ns. The B-DNA and all hydrogen base pairs are preserved, as illustrated in Figure 4. In Figure 10, the 5th base pair structures (on the bottom figure panel) where the azophenyl is inserted, the hydrogen bonding is observed to be preserved even between the base pairs that contain the azobiphenyl adduct. In the top figure, the azobiphenyl adducts fit into the minor groove of the DNA, causing little disruption to the helix base pair stacking and interactions. However, Figure 7 (black line) illustrates that the value for the helix twist for the azobiphenyl DNA adduct significantly differs from the B-DNA dodecamer near base pair 7. The tilt of the damaged DNA adducts also differs significantly from the undamaged helix, as do the slide, shift, and stagger parameters.

The azobiphenyl exhibits significant deviation from B-DNA helix and *anti* glycosidic bonding. According to Figure 5, the 3G and 4A χ angles on one strand are −88.3° and −77.9°, respectively and the 4T and on the second strand is −87.8°. The 4AG/CT and 9TC/GA base pairs no longer exhibit bonding characteristics of B-DNA helix, and this observation is fairly consistent throughout the MD simulation trajectory; the 4AG/CT base pairing and helical structures are completely eliminated by the adduct from the beginning minimized structure, and structures at 50, 60, 70, 80, 90, and 100 ns all exhibit a damaged helix without base pairing in this region. The χ values on the 3G or 4T of one or both strands are consistently representative of *syn* glycosidic bonding. Some of the end values for the χ angles for azophenyl (−62.5°) fall into the *syn* glycosidic range; however, as mentioned previously, there are often end effects and distortion in the end base pairs during the MD simulations of DNA.

In Figure 7 (green bars), values for the GC/GC and CG/CG base pairs near the adduct are significantly different for the azobiphenyl adduct. Values for the roll, twist, slide, rise, shift, propeller, buckle, and stagger differ at the 5, 6, 7, and 8GC from the free DNA dodecamer, indicative of significant conformation perturbation to the DNA structure. In the heat map given in Figure 6, there is a significant difference observed in the major groove of the 4th base pair (adjacent to the adduct). No significant differences are observed over the MD simulation trajectory in χ1 or χ2 angles, opening or stretch parameters. Large differences are observed in the buckle parameter in the adduct from the beginning of the helix through the base pair with the lesion, but no differences are observed in stagger, shear, or shift. Large differences are observed in propeller, roll, twist, and rise in the azobiphenyl adduct around the lesion base pair.

### Changes to DNA Structure With the 4-Aminobiphenyl Adduct

The NMR structure of 4-aminobiphenyl bound to G was solved in 1992 (Cho et al., 1992). This published NMR study presented the conformational perturbation due to the presence of a single dG-C8-ABP adduct (8-([1,1′-Biphenyl]-4-ylamino)-2′-deoxy-guanosine-d5; N-(2′-Deoxyguanosin-8-yl)-4-amino (biphenyl-2′,3′,4′,5′-d5)) in a 15 residue DNA oligomer sequence. However, the sequence of the DNA oligomer differs from the one that we used in this modeling study. In the NMR study, 4-aminobiphenyl C8 substituted dG adducts form two distinct conformations that co-exist in a slow to intermediate exchange rate. One adduct conformation that was labeled “B type” was in the major groove of the helix, the “S conformation” (stacked) displaces the base, and the glycosyidic linkage is modified into the *syn* conformation. The predominant conformation (present in 90%) was the one in the major groove. This observation validated previous modeling predictions (Shapiro et al., 1986) which predicted that the dG-C8-ABP adduct would reside in the major groove and that the aminobiphenyl adduct could be accommodated without significant perturbation to the double-helical structure. These simulations also indicated that the double helix structure was intact with the G in the major groove conformer with hydrogen bonds maintained to the C base pair.

**FIGURE 11:**
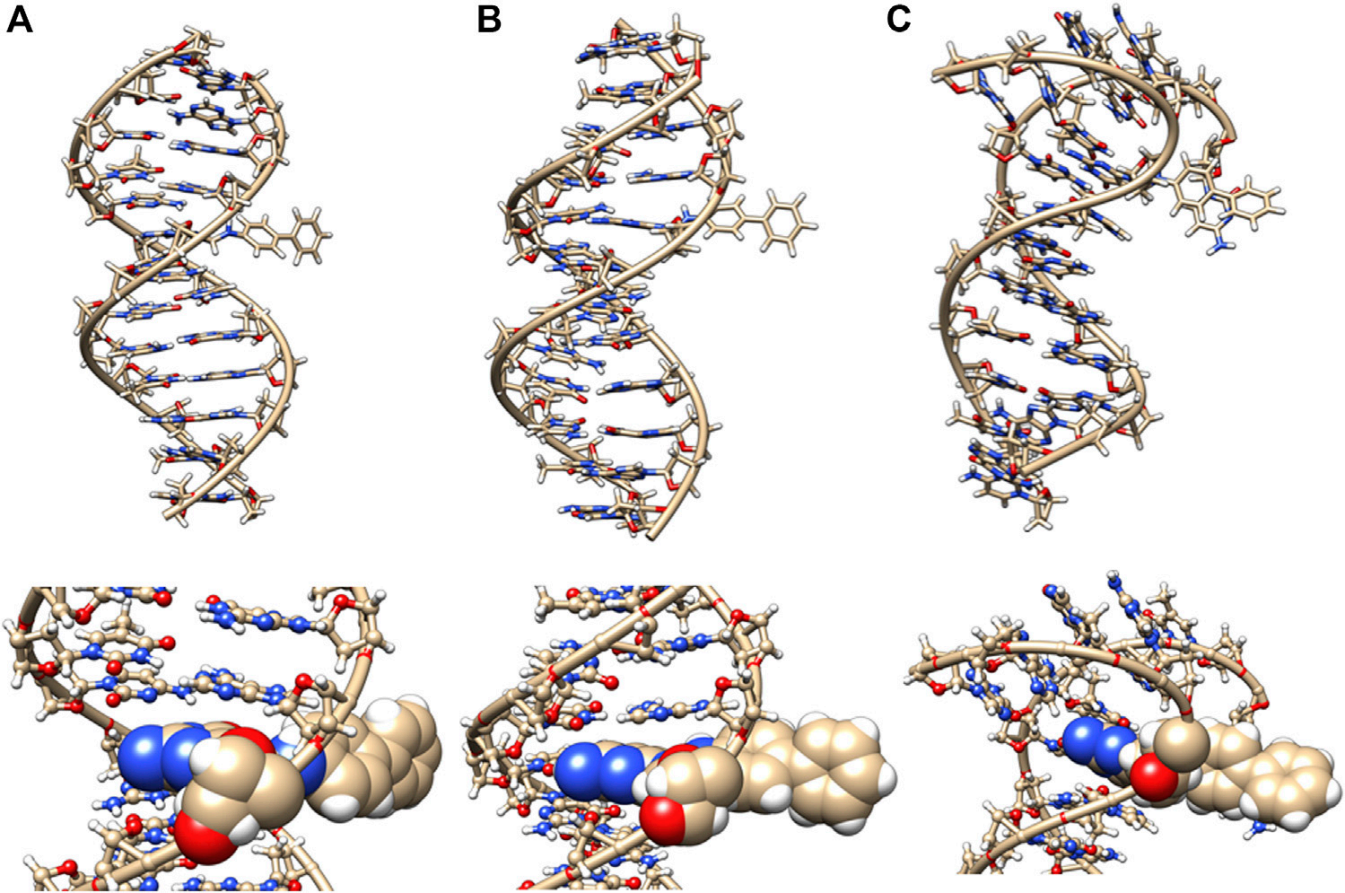
DNA dodecamer with 4-aminobiphenyl dye adduct. **(A)** initial minimized structure, **(B)** at 100 ns of MD simulation, and **(C)** at 1 µs. The bottom row is a zoomed-in version of the adduct region. Note that the helix has completely unwound from the top at 1 µs of MD.

In our MD simulations, the starting minimized structure (Figure 11) was a B helix with the exception of the 4AC/GT base-pair adjacent to where the aminobiphenyl is bound. After 80 ns, the helix is completely destroyed around the 3rd base pair. Only ten pairs of residues are base-paired in the dodecamer. 9C on strand 1 has a χ angle of less than −90°, indicating *syn* conformation after 100 ns, as are 5C on strand 1 and 6C on strand II after 80 ns (Jain et al., 2013). The aminobiphenyl helix exhibits significant variation in χ angles (Figure 5, blue bars) from values characteristic of *anti*-glyosidic bonding with χ values of 56 degrees and −52.2° for the first two residues on the first strand. In Figure 7 (blue bars), values for the GC/GC and CG/CG base pairs near the adduct are significantly different for the 4-aminobiphenyl adduct. Values for the roll parameter differ significantly at AG/CT near the adduct. Stretch for 3GC and 5GC are also significantly different for 4-aminobiphenyl. For propeller, it differs at 3, 4, 7, and 8GC (Jain et al., 2013; Szulik et al., 2013). As observed in Figure 11C, by the end of the 1 µs trajectory, the entire B-DNA helix is disrupted from the top of the helix to the lesion and begins to unfold and collapse. This was the most extreme damage observed for all lesions.

The heat map given in Figure 5 for 4-aminobiphenyl indicates that the major groove was significantly perturbed over the course of the simulation trajectory in the base pairs 2-5 above the DNA lesion damage; the distance over 20 Å compared to less than 20 Å for adduct-free DNA. The minor groove χ1 and χ2 angles were unchanged. Other helix parameters, including opening, stretch, buckle, stagger, shear, shift, propeller, tilt, roll, and rise from the top of the helix to the middle of the helix were completely perturbed over the course of the MD simulation trajectory.

### Free Energy of Binding

The molecular mechanics calculations combined with generalized Born and surface area continuum solvation (MM/GBSA) calculations are widely used for predicting relative binding free energies for congeneric series of ligands (Mulakala and Viswanadhan, 2013). In the present study, we used it to estimate the free energy of interactions between the two strands of DNA. Although these calculations yield approximate values for energies and not absolute measures for free energy of interactions, they provide a reasonable gauge for measuring the relative strength of interactions between the two strands. In other words, this tool can be used as a comparative assessment for energies and stabilities for DNA adducts relative to adduct-free DNA. Therefore, we first obtained the interaction free energy between the two strands of the unaltered DNA as a reference so that the values evaluated for adducts could be directly compared to that while assessing the stability of the DNA double helix due to adduct formation.

Interestingly, Table 1 displays that the Sudan I adduct has a quite comparable average interaction energy as the unaltered system. The free energies for the adduct-free DNA system vary between 100 and 106 kcal/mol, indicating a higher degree of freedom in making and breaking interactions within the two strands. However, except for the two extreme energy values (104 and 99 kcal/mol) of the Sudan I adduct, the range of energies of the other eight samples is only 1 kcal/mol, indicating some loss of freedom in mobility. Meanwhile, for Sudan II, its larger negative values indicate an increased strength in the strand interaction. However, the azobiphenyl adduct had a slightly reduced free energy of interaction as compared with the unaltered DNA, while a significant reduction in the free energy was observed for the aminobiphenyl adduct. All ten samples selected at 100 ns segments of the trajectory of the aminobiphenyl adduct also have the same reduced strength of interaction. As indicated in Figures 8–11, the aminobiphenyl adduct is observed to have the most disrupted helical conformation, and that is rather well captured in the value of the reduced free energy of interaction between the strands.

**TABLE 1:** Free energies (in kcal/mol) of interaction between the two strands estimated from MM/GBSA calculations for each DNA adduct. The unaltered DNA system is used as the reference. A total of 100 configurations collected at each nanosecond from 100 ns segments of each µs trajectory is considered as a sample, and the MMGBSA energies (and the standard errors) tabulated were averaged from the interaction energies of 100 configurations per sample. The average energies and standard errors given in the last row were calculated from those ten samples.

Sample	DNA		Sudan I adduct		Sudan II adduct		Azobiphenyl adduct		4-Aminobiphenyl adduct	
	Energy	Std. error	Energy	Std. Error	Energy	Std. Error	Energy	Std. Error	Energy	Std. Error
1	−106.39	0.56	−103.65	0.54	−105.41	0.53	−104.34	0.64	−95.81	0.74
2	−106.60	0.56	−103.77	0.54	−107.05	0.51	−102.95	0.67	−96.29	0.91
3	−101.36	0.62	−103.37	0.54	−103.29	0.67	−102.25	0.62	−97.16	0.65
4	−100.54	0.69	−103.44	0.60	−105.48	0.55	−98.00	0.67	−86.84	0.79
5	−101.78	0.65	−103.16	0.50	−106.25	0.55	−100.40	0.71	−96.41	0.72
6	−104.81	0.64	−99.36	0.67	−106.54	0.54	−98.11	0.65	−94.92	0.66
7	−106.89	0.58	−104.21	0.55	−106.61	0.48	−103.42	0.69	−99.85	0.98
8	−100.83	0.64	−103.87	0.56	−106.82	0.54	−102.98	0.58	−100.07	0.72
9	−102.16	0.68	−103.69	0.57	−106.22	0.52	−104.05	0.62	−91.19	1.01
10	−102.17	0.74	−103.41	0.59	−104.94	0.56	−102.14	0.70	−84.50	0.72
average	−103.35	0.80	−103.19	0.44	−105.86	0.36	−101.86	0.72	−94.31	1.65

## Discussion

Understanding the structural changes in DNA conformations due to the presence of dye and dye-metabolite adducts is the key to understanding their biological impact. This knowledge of the molecular mechanism will lead to approaches to develop, control, or minimize biological damage to DNA due to environmental exposure to potential dye and dye metabolite toxins and mutagens (Gauvin and Broyde, 2001). Much of DNA damage arises from the addition of polyaromatic hydrocarbons or heterocyclic amines to C8, N6, N2, or N7 purine bases in DNA. This is primarily caused by environmental pollutants such as dyes, tobacco, pesticides, food mutagens, smoke, and other toxins.

In this study, we determined that each of the adducts caused significant perturbation to the DNA helical structure and conformation. In the case of Sudan II, the large adduct causes a significant increase in the free energy of interactions between the two strands due to its exposure and extrahelical extension. In the starting minimized structure of the DNA dodecamer with the Sudan II adduct, the B-DNA helix and hydrogen bonding are disrupted, and the phosphate to phosphate major groove distances are larger than the free dodecamer helix. After 100 ns of MD simulations, all Watson–Crick base pairing and hydrogen bonding within the helix is maintained, and phosphate to phosphate major grove distances for the base pairs around the adducts (the 4th and 5th base pairs) are larger than the unaltered DNA. The Sudan I bound DNA adduct also exhibited larger phosphate to phosphate groove distances than the unaltered DNA around the adduct (4th base pair). The overall distortion in the DNA helix due to adjacent base pair angle distortions from the insertion of the Sudan adducts in the DNA is reflected by larger values for twist, roll, and tilt. Damage due to the DNA helix is significant with the 4-aminobiphenyl adduct in the DNA dodecamer observed after 100 ns of MD simulations; both Watson–Crick base pairing and the double-stranded helix form are not maintained. There are no shift, slide, rise, tilt, roll, or twist parameters for the 5th GC base pair, and no phosphate to phosphate major groove or minor groove distances are reported surrounding the lesion. In the case of the azobiphenyl adduct linked to deoxyguanosine dG-C2 in a DNA dodecamer, after 100 ns of MD simulations, the Watson–Crick base pairing and hydrogen bonds are preserved. However, there is a significant difference in tilt and roll parameters around the lesion, indicating deformation between 2 base pair planes along the long and short base pair axis. The phosphate to phosphate major groove around the lesion is ∼2–3 Å larger.

There are a variety of mechanisms to repair DNA adducts and lesions, including base excision repair (BER) and nucleotide excision repair (NER). BER removes specific non-bulky lesions, while NER removes larger bulky lesions that often cause DNA distortions and destabilizations, so more drastic DNA repair mechanisms are required as opposed to simply removing the chemically damaged base. DNA duplexes damaged by polycyclic aromatic hydrocarbons, such as described by the lesions in these studies, are repaired by NER. Often damage caused by environmental toxins is resistant to DNA repair mechanisms, which results in cellular damage and can lead to carcinogenesis. DNA conformational changes can also cause a loss in recognition or difficulty in replication of the damaged DNA by polymerases and the enzymes involved in the repair process. The conformation of the DNA adduct affects the repair process by NER. The amount of local destabilization induced by a lesion regulates how efficiently it is recognized and repaired (Geacintov and Broyde, 2017). Usually, large bulky lesions/adducts in DNA, such as the ones caused by the Sudan dyes, are recognized and repaired by NER pathways. Distortions to the local B-DNA environment control the mechanism of repair; NER recognizes only the local distortions to DNA and not the lesion itself. Adduct damages cause structural changes not only at the lesion site but also at flanking base pairs (Lukin and de los Santos, 2006).

Sudan dyes are environmental agents that form bulky adducts with DNA. Conformational properties of adduct-dG pairs are known to affect the efficiency of the nucleotide excision repair process. Much less is known about N-bonded adducts to DNA than C8 bonded adducts to G, as these are the most common. Some conformational search algorithms have been developed to assess the effects of 3′ versus 5’ modification of bases with adducts; interactions of the adducts with neighboring bases and sugars cause different conformation preferences for the adducted bases. Several molecular modeling studies with polymerases have suggested that polymerases with large active sites can accommodate bulky adducts, which results in these polymerases bypassing the lesions. Bypassing the lesions can result in errors in DNA replication, which results in mutational errors.

DNA polymerases recognize sites to repair by scanning and recognizing the DNA minor groove, a process called “minor groove scanning”, and it is influenced by the hydrogen bonds between the minor groove site of the nucleobases and the enzyme. In solved ternary structures of DNA polymerases with DNA and dNTP substrates, O2 atoms of pyrimidines and N3 atoms of purines in the minor grove nucleobases interact with the side chains of polymerase proteins directly or *via* water molecules. Minor groove disruptions can lead to the inability to align and interact with these components and prevent DNA repair catalysis from occurring. DNA cannot be extended or elongated without a hydrogen bonding interaction between the primer-terminal nucleobase and the polymerase enzyme (McCain et al., 2005; Ludmann and Marx, 2016).

Molecular modeling studies of DNA with adducts can be valuable because they provide an avenue for making valid predictions that complement experiments. For example, MD simulations correctly predicted a G-G mismatch when there is a C-linked phenoxy adduct to the G, which causes Hoogsteen base pairing instead of the standard B DNA Watson–Crick base pairing and leads to a G to C mutation when the DNA is replicated (Millen et al., 2012). MD simulations can often provide dynamical structure information and details that are not accessible from static X-ray or NMR structures. Understanding the interaction of DNA-adduct lesions with polymerase active sites can result in a complete model for mechanisms of mutagenesis and genotoxicity. For example, from combined experimental and modeling studies with an 11-mer DNA duplex [d (50-CCATCG*CNACC-30).d (50-GGTNGCGATGG-30)] in which G* is a lesion with a polycyclic aromatic adduct, it was possible to examine the NER excision repair process in the *E. Coli* UvrABC assay (Jain et al., 2013). It was reported that the lesion-induced DNA bending was the most significant factor affecting repair, complementing the experimental studies.

In undamaged DNA, purines A and G base pair, respectively, with pyrimidines T and C *via* Watson–Crick hydrogen bonds. Watson–Crick base pairing requires the bases to have an *anti*-conformation around the glycosidic bond. When the base adopts the *syn* conformation, Hoogsteen base pairing occurs, which results in mutations. DNA sequence length and sequence context can influence whether there is a *syn* vs. *anti*-glycosidic bond. Single adducts can lead to several mutations depending on the DNA sequence content. We chose this particular DNA dodecamer for the study with adducts; however, different DNA sequences and DNA lengths may lead to different conformational results. Conformation of a bulky adduct can lead to a significant alteration in the DNA helix structure, as illustrated by our study.

The C8-site of 2′-deoxyguanosine (dG) is the most common site for most DNA adducts to bind DNA and cause carcinogenesis. C8-dG adducts are classified into three categories in the major groove called B-type, the base-displaced stacked (S-type), and the minor groove wedge (W-type). Whether the B, W, or S conformation is formed also has an effect on how efficiently the damage caused by the lesion is repaired. These have been characterized using NMR spectroscopy, and the DNA conformation type is dependent on the nature of the attached C8-moiety and the DNA sequence (Millen et al., 2012; Sproviero et al., 2015). Environmental chemicals can modify histones and alter DNA methylation indirectly by acting on enzymes that modify DNA or through oxidative stress; for example, benzene can promote hypomethylation of LINE1 and Alu repetitive elements and hypermethylation of tumor suppressor gene p15. Changes in epigenomes are found in many cancers. Polycyclic aromatic hydrocarbons can cause epigenetic alterations; exposure to polycyclic aromatic hydrocarbons causes a loss in DNA methylation in murine embryonic fibroblast cells. The binding of *anti* (þ)*anti*-benzo [a]pyrene-trans-7,8- dihydriol-9,10-epoxide (BPDE) to G in 5-MeCpGs is associated with mutation adducts formed on 5mecpGs, which affects its conformation and are removed less efficiently (Swagatika and Tomar, 2016). Thus, the present study indicates that in systems that modify deoxyguanosine residues, there could potentially be an impact on the epigenome based on the binding of these guanosine nucleotides to methylated cytosines.

## Conclusions

Starting with the possible G adducts suggested from NMR and MS experiments reported in the literature, we have performed MD simulations for DNA adducts of 2 azo dyes and 2 metabolites. The specific goal of the present study was to gain an understanding of the possible molecular mechanisms of azo dye toxicity through the changes in structure and dynamics due to the formation of DNA adducts. The unaltered reference DNA dodecamer structure maintained its B-DNA double helix form and base pair hydrogen bonding during the entire MD simulation trajectory. However, the 5-dG Sudan I and Sudan II DNA adducts were demonstrated from these simulations to disturb the Watson Crick base pairing and hydrogen bonding with significant DNA helix distortions. Although the azobiphenyl adduct may fit nicely into the DNA minor groove, it too still causes significant deviations from the B-DNA helix form. The aminobiphenyl adduct found in the major groove was observed from these simulations to cause the most extreme damage to the helical structure of the DNA adduct. When compared with the ligand-free DNA, the changes in free energy of interactions between the two DNA strands indicated that the smaller and buried azobiphenyl and aminobiphenyl adducts may weaken the helical structure, whereas the Sudan II adduct increases the strand interactions of DNA.

The molecular modeling initiated in this work can potentially serve as a guide to estimate the damage to DNA caused by Sudan azo dyes and azo dye metabolites due to adduct formation with DNA. The results, at the molecular level, help us evaluate structural consequences due to DNA adduct formation that may ultimately lead to mutagenesis and carcinogenesis. Also, molecular details resulting from the simulations can provide an improved understanding of mutagenic agents and can assist in guiding the design of less genotoxic dyes.

## Data Availability

The raw data supporting the conclusions of this article will be made available by the authors, without undue reservation.
