# Supplementary material for: Molecular Modeling Study of the Genotoxicity of the Sudan I and Sudan II Azo Dyes and Their Metabolites
Source: Front Chem. 2022 Jun 23;10:880782. doi: 10.3389/fchem.2022.880782 (PMC9261194; doi:10.3389/fchem.2022.880782)
Supplement: Supplementary file 1 [file DataSheet2.pdf]

# Molecular Modeling Study of the Genotoxicity of the Sudan I and Sudan II Azo Dyes and Their Metabolites

**Rachelle J. Bienstock[1,2], Lalith Perera[2,*], and Melissa A. Pasquinelli[1,3*]**

[1] Fiber and Polymer Science Program, Wilson College of Textiles, North Carolina State University, Raleigh, NC, United States

[2] National Institute of Environmental Health Sciences, Research Triangle Park, Durham, NC, United States

[3] Forest Biomaterials, College of Natural Resources, North Carolina State University, Raleigh, NC, United States

**Supplementary Materials**

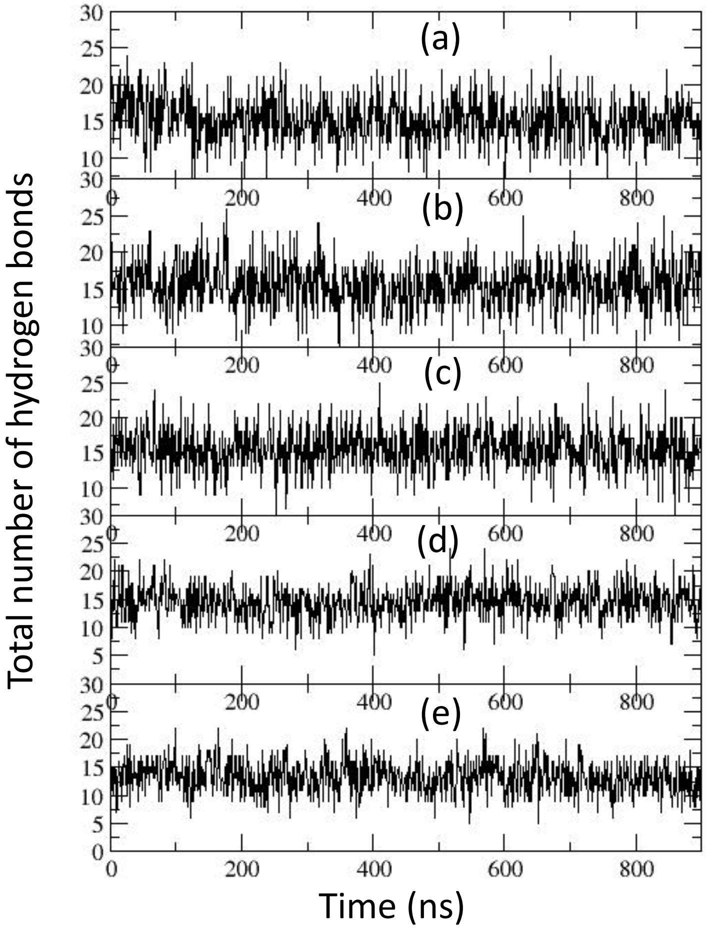


**Figure S1**: The change in the overall number of hydrogen bonds over the 1 μs trajectory. Panel A: unadducted DNA; Panel B: DNA with Sudan dye adduct; Panel C; DNA with Sudan II dye adduct; Panel D: DNA with azobiphenyl adduct; Panel E: DNA with 4-aminobiphenyl adduct.

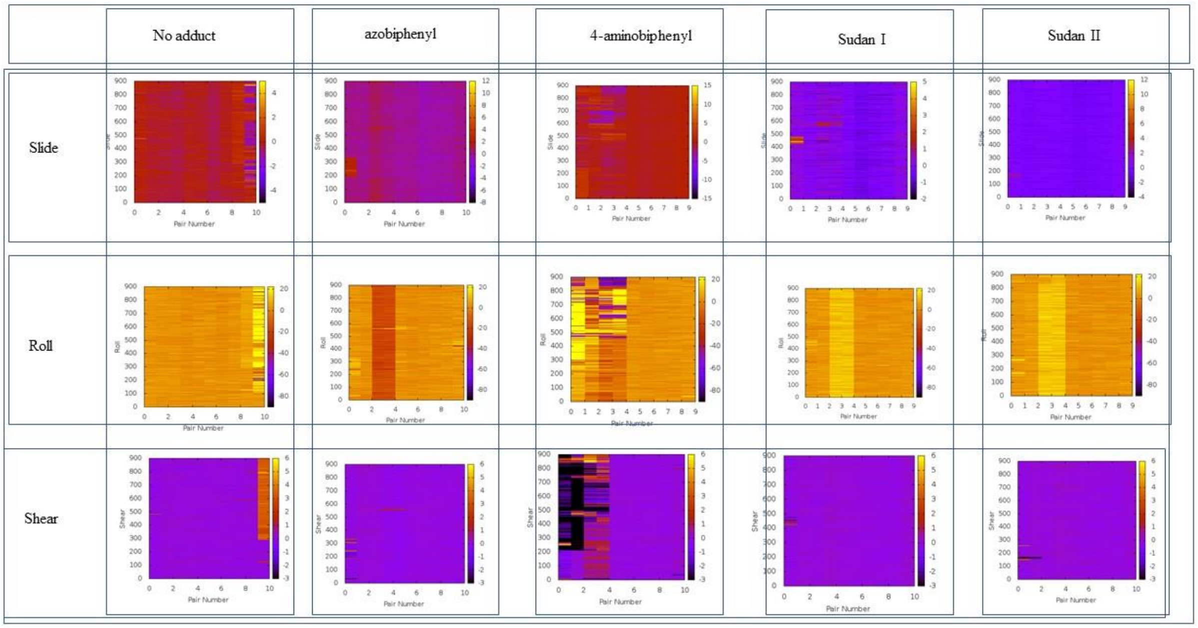
No adduct
azobiphenyl
4-aminobiphenyl
Sudan I
Sudan II
Slide
Roll
Shear
Pair Number

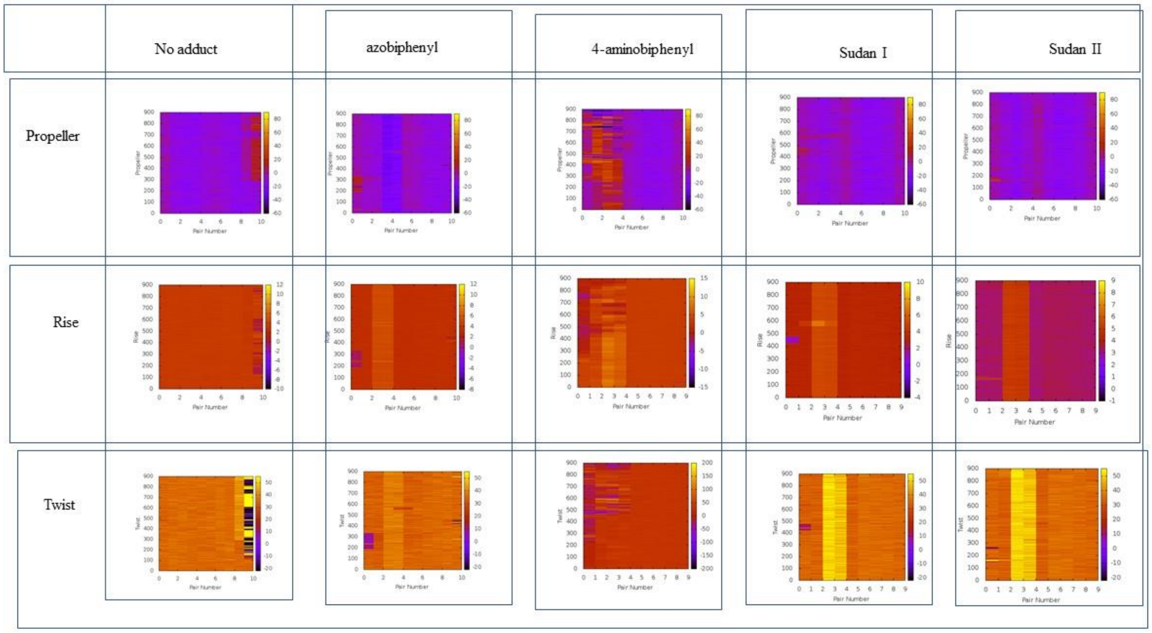
No adduct
azobiphenyl
4-aminobiphenyl
Sudan I
Sudan II
Propeller
Rise
Twist
Pair Number

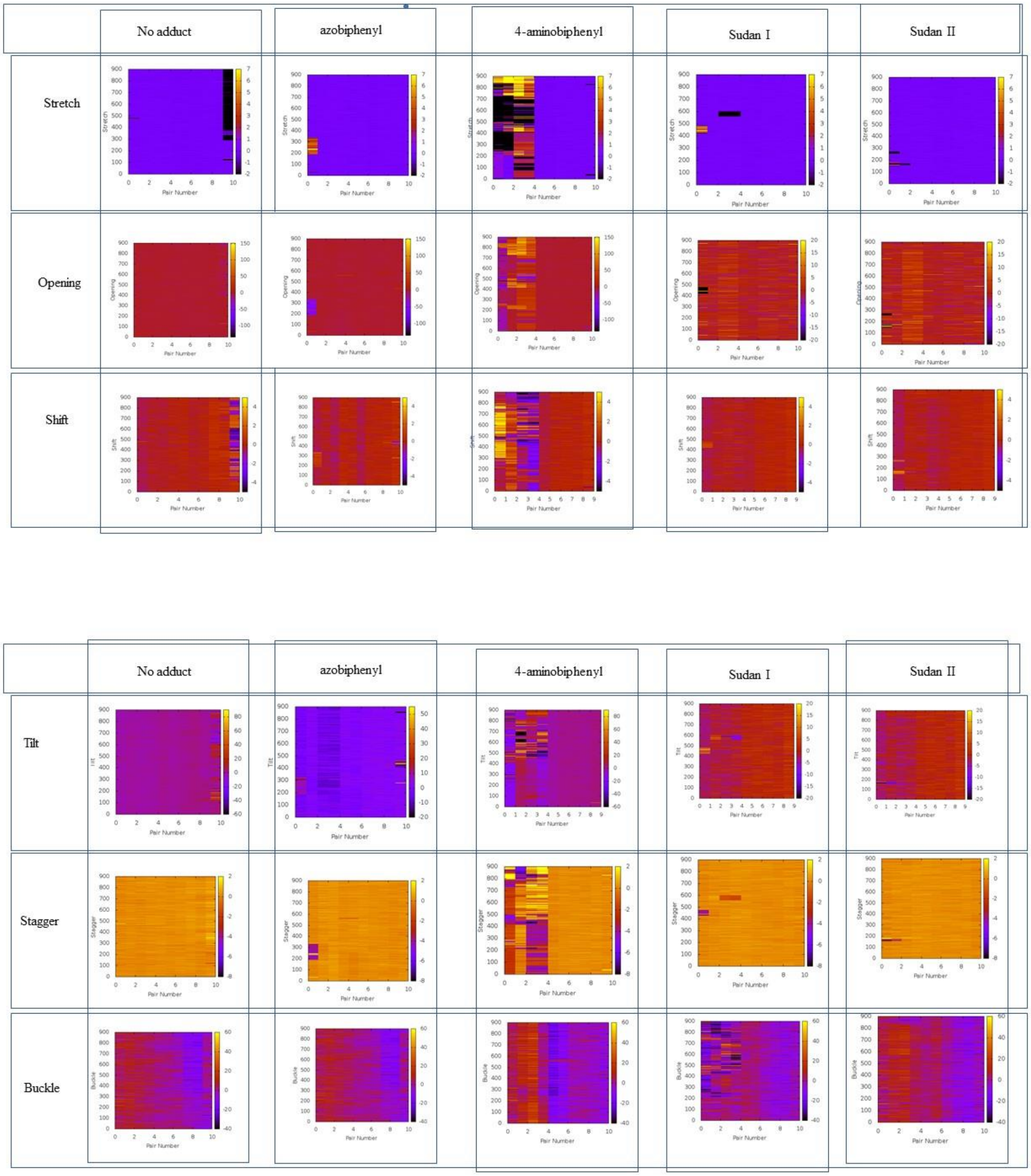


**Figure S2**. Heat Maps of 3DNA parameters changing over the course of the 1 μs MD trajectories of Sudan I and II, azobiphenyl, and 4-aminobiphenyl adducted DNA.
